# UHPLC-MS/MS Quantification Combined with Chemometrics for Comparative Analysis of Different Batches of Raw, Wine-Processed, and Salt-Processed Radix *Achyranthis Bidentatae*

**DOI:** 10.3390/molecules23040758

**Published:** 2018-03-26

**Authors:** Liu Yang, Hai Jiang, Meiling Yan, Xudong Xing, Xinyue Guo, Bingyou Yang, Qiuhong Wang, Haixue Kuang

**Affiliations:** 1 Key Laboratory of Chinese Materia Medica, Heilongjiang University of Chinese Medicine, Ministry of Education, Harbin 150040, China; hxk_yl@163.com (L.Y.); jianghai_777@126.com (H.J.); hxk_yan@163.com (M.Y.); mrxing_xudong@126.com (X.X.); m17645028606@163.com (X.G.); ybywater@163.com (B.Y.); 2 School of Traditional Chinese Medicine, Guangdong Pharmaceutical University, Guangzhou 528458, China

**Keywords:** Radix *Achyranthis bidentatae*, UHPLC–MS/MS, processing, quality assessment, chemometrics

## Abstract

An accurate and reliable method using ultra-high performance liquid chromatography combined with triple quadrupole tandem mass spectrometry (UHPLC–MS/MS) was established for simultaneous quantification of five major bioactive analytes in raw, wine-processed, and salt-processed Radix *Achyranthis bidentatae* (RAB). The results showed that this method exhibited desirable sensitivity, precision, stability, and repeatability. The overall intra-day and inter-day variations (RSD) were in the range of 1.57–2.46 and 1.51–3.00%, respectively. The overall recoveries were 98.58–101.48% with a relative standard deviation (RSD) of 0.01–1.86%. In addition, the developed approach was applied to 21 batches of raw, wine-processed, and salt-processed samples of RAB. Hierarchical clustering analysis (HCA), principal component analysis (PCA), heat map, and boxplot analysis were performed to evaluate the quality of raw, wine-processed, and salt-processed RAB collected from different regions. The chemometrics combined with the quantitative analysis based on UHPLC–MS/MS results indicated that the content of five analytes increased significantly in processed RAB compared to raw RAB.

## 1. Introduction

Herb processing of herbal medicines, including the special crafts of steaming, baking, decocting, and other methods with liquid or solid Supplementary Materials, plays an important role in the application of traditional Chinese medicine (TCM). There is a close relationship between processing, quality, and efficacy of herbal medicines. The discrimination between raw and processed herbal medicines is a basic and important task for the investigation of the mechanism of herb processing and the quality control (QC) of herbs.

Radix *Achyranthis bidentatae* (RAB), derived from the dried roots of *Achyranthes bidentata* Bl., has been used in traditional Chinese medicines for nourishing the liver and kidney, strengthening bones and tendons, promoting diuresis, relieving dysuria and promoting blood circulation [1,2,3,4,5,6,7,8,9,10]. Generally, TCM should be processed before clinical use. RAB has been used in three forms in TCM: one is raw RAB (RRAB), while the other two are processed with the wine and salt method, which is called wine RAB (WRAB) and salt RAB (SRAB), respectively. According to the “Shen Nong’s Herbal Classic” records, RAB needs to be processed before clinical use in helping the kidney and strengthening bones, muscles, and tendons. Particularly, after being processed with salt, the promotion of blood circulation and restoration of bone fractures will be strengthened [11,12]. Several types of chemical analytes, particularly phytoecdysones and triterpenoid saponins, have been identified from the roots of RAB [13,14,15,16,17,18,19,20]. Lei et al. [21] showed that *β*-ecdysterone prevented the process of osteoporosis. Gao et al. [22] demonstrated that *β*-ecdysterone promoted the proliferation of osteoid cells UMR106. Dong et al. [2] revealed its effects on treatment of osteoporosis in ovariectomized rats. Yu et al. [6] demonstrated that triterpenoidal saponins could inhibit osteoclast formation and thus, could be used as bone resorption inhibitors to treat osteoporosis. Guo et al. [8] suggested that *Achyranthes bidentata* Saponins (ABS) stimulated osteogenic differentiation of bone mesenchymal stem cells (BMSCs) via activation of the extracellular regulated protein kinases (ERK) signaling pathway. Ren et al. [23,24] demonstrated that ABS improved bone metabolism of osteoporosis induced by retinoic acid in rats. Therefore, RAB was used for nourishing liver and kidney as well as strengthening bones and tendons due to the interaction of multiple components. However, for raw, wine-processed, and salt-processed RAB, it is well known that a single marker compound (*β*-ecdysterone) might not accurately reflect the intrinsic quality control and response for the overall pharmacological activities of the complex herbal product. Thus, it is necessary to develop a reliable method for simultaneous quantification of five bioactive analytes, including *β*-ecdysterone, 25-S inokosterone, 25-R inokosterone, ginsenoside R_0_, and chikusetsusaponin Iva, for quality control of raw, wine-processed, and salt-processed RAB.

Various studies [25,26,27] have focused on the phytoecdysones and triterpenoid saponins of RAB using high performance liquid chromatography (HPLC) with diode array detection and UHPLC coupled with evaporative light scattering detector (ELSD). However, the methods resulted in waste solvent, long analysis time, and poor selectivity with only retention time through the identification of chromatographic peaks, which was generally not adequate for the analysis of herbal samples characterized by the complex matrix. Although some methods based on HPLC have been reported for the determination of several of these components in RAB, there is still no method established for the simultaneous determination of the phytoecdysones and triterpenoid saponins using UHPLC–MS/MS. Furthermore, no report is available on comparative studies of the processing methods for these analytes. The UHPLC–MS/MS technique has the advantages of abundant mass fragmentations and many scan modes afforded by tandem mass spectrometry, which can provide the required specificity and sensitivity as well as decrease separation time and solvent consumption. Therefore, this method is appropriate for simultaneous determination of the polarity differences of the two types of target analytes and the existence of isomers (*β*-ecdysterone, 25-R inokosterone, and 25-S inokosterone). 

The traditional uses of RAB have been largely expanded upon. Experimental studies indicate that RAB possesses a number of pharmacological activities, including anti-tumor [28], immunostimulant [29,30], anti-fertility [31,32], anti-bacterial [33], anti-inflammatory [34], cognition-enhancing [35], anti-senile [36,37], antioxidant [38,39], and anti-osteoporosis [40,24,2] properties. In terms of treatment, RAB has been used to influence carbohydrate metabolism in the blood [41,42], hasten growth [43], and improve the dual modulatory function of the immune system [44,45]. Therefore, RAB has received considerable critical attention. However, our research has shown differences in the content of different batches of Chinese medicine from the Henan Province. This might be due to some factors, such as intrinsic factors, including plant region and genetic variation, and extrinsic factors, including the season (different climatic), geography (soil and minerals conditions), harvest time, storage conditions, and environmental pollution [46,47,48]. To explore these complex factors of RAB, UHPLC-MS/MS combined with chemometric tools is the preferred method of sample comparison for quality assessment requirements. This method is increasingly important in the quality assessment of herbal medicines. 

In this study, an UHPLC–MS/MS method was established for simultaneous quantification of five bioactive analytes, including *β*-ecdysterone, 25-R inokosterone, 25-S inokosterone, ginsenoside R_0_, and chikusetsusaponin IVa in raw, wine-processed, and salt-processed RAB. The validated method was applied to evaluate the quality of the samples from different batches and processed methods, before the results were further analyzed by hierarchical clustering analysis (HCA), principal component analysis (PCA), heat map, and boxplot analysis to provide more information about the differences in each sample. 

## 2. Results

### 2.1. Optimization of Extraction Conditions 

Three parameters, including extraction time (30, 40, 60, and 80 min), solvent volume (25, 50, and 75 mL) and extraction solvent (25, 50, 75, and 100% methanol/water), which may prejudice the extraction efficiency of ultrasonication, were optimized using univariate analytes. The extraction rates of the five major analytes were gradually increased as the extraction time was increased from 10 to 80 min. Further increases in the extraction time did not increase the extraction rates of the five major analytes. Thus, 60 min was adequate for the extraction procedure. Secondly, the effect of solvent volume was of great importance for the extraction procedure. When solvent volume was increased from 25 to 50 mL, the extraction rates of the five major analytes gradually increased. When the solvent volume was increased to 75 mL, the extraction rates of the five major analytes decreased. This phenomenon may be due to the dilution effect exceeding the hydrotropic effect of increased solvent. Thus, 50 mL of solvent volume was enough for the extraction of the five major analytes. Thirdly, the solvent ratio between methanol and water that may also bias the methanol ratio was increased from 25, 50, 75, and 100%. The largest extraction rates of the five major analytes were achieved by using a solvent ratio of 50% (Figure S1). Finally, the optimal conditions for the extraction procedure were as follows: solvent volume of 50 mL; extraction time of 60 min; and solvent ratio of 50% methanol/water. 

### 2.2. Optimization of Chromatographic and Mass Spectrometric Conditions

**Figure 1:**
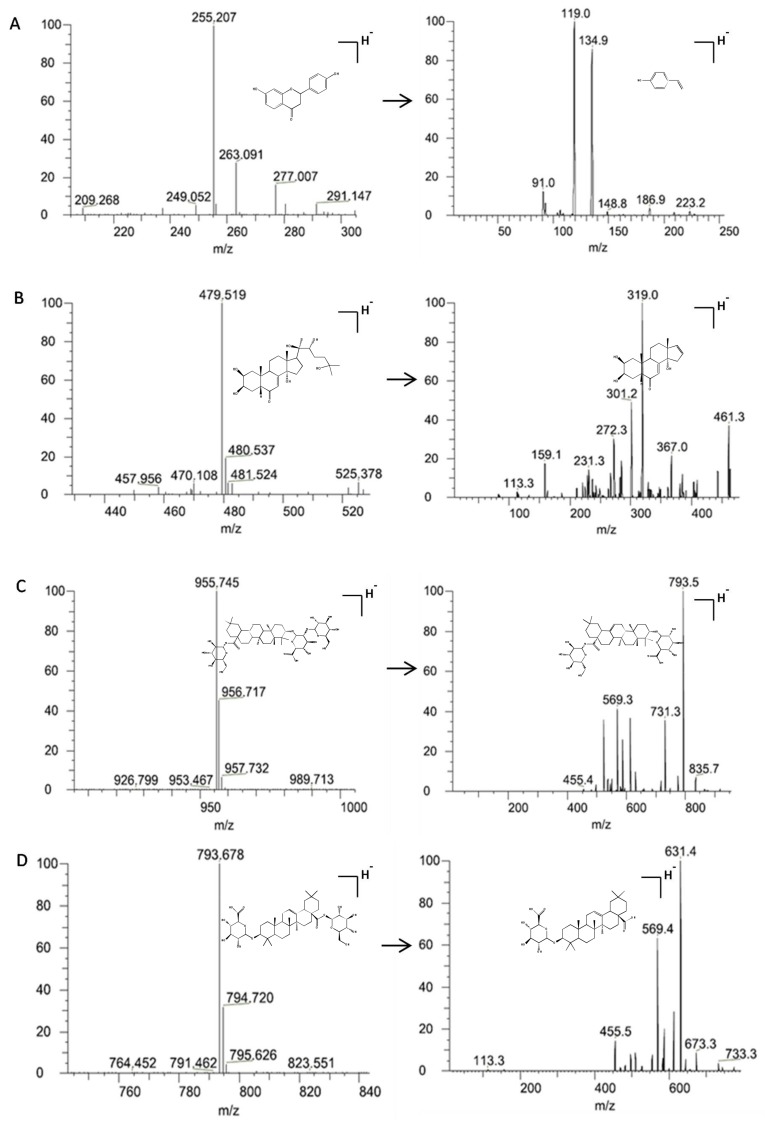
Representative MS/MS spectra of glycyrrhizin (IS) (**A**), *β*-ecdysterone, 25-R inokosterone, 25-S inokosterone, (**B**) ginsenoside R_0_ (**C**), and chikusetsusaponin IVa (**D**). Among them, there are isomers (*β*-ecdysterone, 25-R inokosterone, and 25-S inokosterone) that have the same *m*/*z* for the precursor and product ions, which only showed the MS/MS spectra of *β*-ecdysterone.

**Figure 2:**
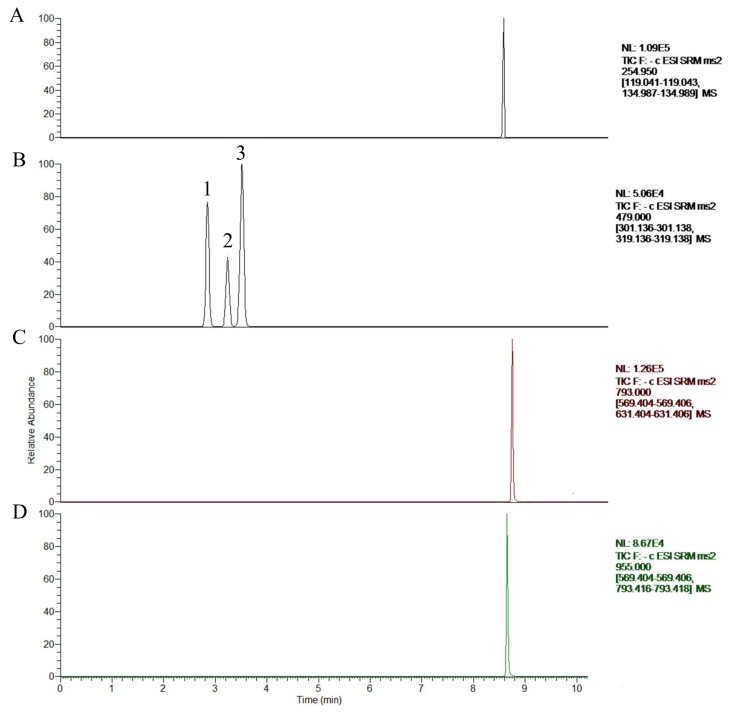
Total ion chromatogram with time scanning of glycyrrhizin (IS) (**A**), *β*-ecdysterone (**B1**), 25-R inokosterone (**B2**), 25-S inokosterone (**B3**), chikusetsusaponin IVa (**C**), and ginsenoside R_0_ (**D**).

Two types of analytes (phytoecdysones and triterpenoid saponins) differ greatly due to their structural features. This is especially the case for the rapid and complete separation of polar phytoecdysones isomers (i.e., *β*-ecdysterone, 25-R inokosterone, and 25-S inokosterone), which have the same *m*/*z* for the precursor and product ions. Hence, mobile phase selection is critical in separating these analytes properly. To achieve optimal separation and peak shape without excessive peak tailing in a short analysis time, the chromatographic conditions, such as mobile phase, solvent modifier, and gradient program, were optimized in the preliminary test. For *β*-ecdysterone, 25-R inokosterone, 25-S inokosterone, ginsenoside R_0_, and chikusetsusaponin Iva, an acetonitrile/water mobile phase system yielded better signal intensity than that of methanol/water. Simultaneously, different concentrations (0.1%, 0.3%, and 0.5%) of acetic acid were checked to optimize ionization. Moreover, the signal intensities of the analytes were investigated by adding acetic acid to the water phase. The addition of 0.3% acetic acid significantly increased the signal intensity of the five analytes, especially for the acetonitrile/water system. Meanwhile, the addition of 0.3% acetic acid was beneficial to the peak shape of the analytes. Satisfactory separation was achieved after 10 min by gradient elution using an acetonitrile/water (0.3% acetic acid) system at a flow rate of 0.3 mL/min. The reference compound of each analyte was directly infused into the mass spectrometer (MS) along with the mobile phase to optimize the MS conditions. The mass responses of the five analytes in both positive and negative modes were investigated, with a better response obtained in the negative ionization mode. Throughout the development of the method, the selectivity, reproducibility, and robustness of the MS method were monitored and adjusted to establish methods that could be validated and applied. The following multiple reaction monitoring modes were found to be specific and intense (Figure 1) for the analysis of *β*-ecdysterone (*m*/*z* 479.519→319.0), ginsenoside R_0_ (*m*/*z* 955.745→793.5), chikusetsusaponin IVa (*m*/*z* 793.678→631.4), and glycyrrhizin (IS) (*m*/*z* 255.207→119.0). The details are presented in Table 1 and Figure 2.

### 2.3. Method Validation

The proposed UHPLC–MS/MS approach for quantitative analysis was validated by determining the linearity, limit of detection (LOD), limit of quantity (LOQ), intra-day and inter-day precisions, stability, repeatability, and accuracy. The results are summarized in Table 2 and Table 3. The method had a good linear range and the results determined were accurate and reliable. The LOD and LOQ were in the range of 0.325–0.499 and 0.955–1.485 mg/mL, respectively. The overall intra-day and inter-day variations (RSDs) were in the range of 1.57–2.46 and 1.51–3.00%, respectively. The repeatability and stability presented as RSD were in the range of 2.16–3.00 and 2.54–2.97%, respectively. The overall recoveries were 98.58–101.48% with RSDs of 0.01–1.86%. These results indicated that the developed UHPLC–MS/MS approach was sensitive, repeatable and accurate for the quantitative analysis of the five analytes.

### 2.4. Quantitative Analysis and Boxplot Analysis of Raw, Wine-Processed, and Salt-Processed Products

RAB is one of the ancient and frequently used herbal medicines in both Eastern and Western countries, with its use dates back more than 2000 years. In TCM, RAB is usually processed and traditionally used to reinforce the muscles and bones, improve the tone of liver and kidneys and promote blood flow. *β*-ecdysterone, 25-R inokosterone, 25-S inokosterone, ginsenoside R_0_, and chikusetsusaponin IVa are the main components of the curative effect of the RAB. Therefore, it is necessary to develop a reliable method for simultaneous quantification of *β*-ecdysterone, 25-R inokosterone, 25-S inokosterone, ginsenoside R_0_, and chikusetsusaponin IVa. The newly developed UHPLC–MS/MS approach was employed for simultaneous quantification of five analytes, including *β*-ecdysterone, 25-R inokosterone, 25-S inokosterone, ginsenoside R_0_, and chikusetsusaponin IVa, in 21 batches of raw, wine-processed, and salt-processed products from different regions of the Henan Province. The quantitative analyses were performed by means of the internal standard method. Each sample was extracted and analyzed, with the analytical results summarized in Table 4. The study found that there were large variations in the contents of the five major analytes in different batches of raw, wine-processed, and salt-processed samples. The results proved that the content of salt-processed RAB is better than that of raw products. The highest content of *β*-ecdysterone was obtained in each sample, followed by ginsenoside R_0_ and chikusetsusaponin IVa. In addition, there are differences in the content of different batches of Chinese medicine from the Henan Province. Therefore, we need to measure these five major analytes to control the quality of the RAB. Meanwhile, salt-processed RAB samples had the highest content of analytes extracted from RAB, which was shown through UHPLC–MS/MS analysis. According to the theories of TCM and the results of this study, the treatment effects of salt-processed RAB are better than that of raw products due to the increase in content of the active ingredients.

**Figure 3:**
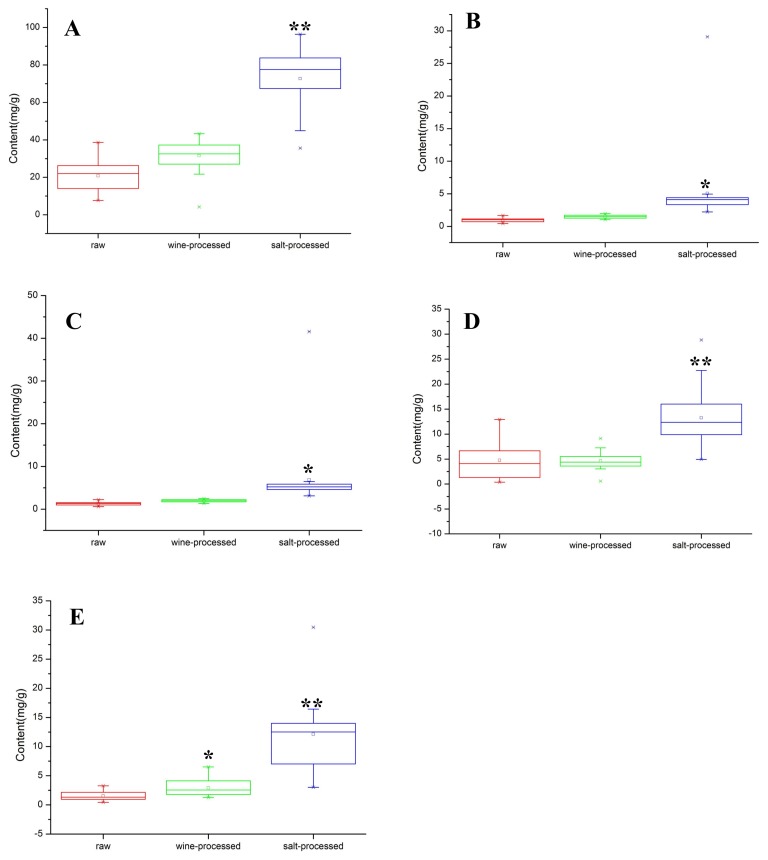
Boxplots of the five target analytes of 21 batches in raw, wine-processed, and salt-processed samples RAB. The **A**–**E** in the figure represents the five major analytes in raw, wine-processed, and salt-processed RAB, including: *β*-ecdysterone (**A**); 25-S inokosterone (**B**); 25-R inokosterone (**C**); ginsenoside R_0_ (**D**); and chikusetsusaponin IVa (**E**). Compared with raw samples, * *p* < 0.05, ** *p* < 0.01; the middle line in the boxplot represents the median.

In this work, boxplot analysis was employed to evaluate the quality of 21 batches through the data distribution of five target analytes. The content of five target analytes in raw, wine-processed, and salt-processed products is shown in Figure 3. From the boxplots, it is clear that *β*-ecdysterone, 25-S inokosterone, 25-R inokosterone, ginsenoside R_0_, and chikusetsusaponin IVa have higher concentrations in salt-processed samples. Otherwise, the most important finding was the content of salt-processed RAB being better than that of raw and wine-processed RAB. It has become necessary to develop a reliable method for simultaneous determination of the concentration of five target analytes, including *β*-ecdysterone, 25-S inokosterone, 25-R inokosterone, ginsenoside R_0_, and chikusetsusaponin IVa, in raw, wine-processed, and salt-processed for quality control of RAB.

### 2.5. Chemometric Analysis

#### 2.5.1. Hierarchical Clustering Analysis

**Figure 4:**
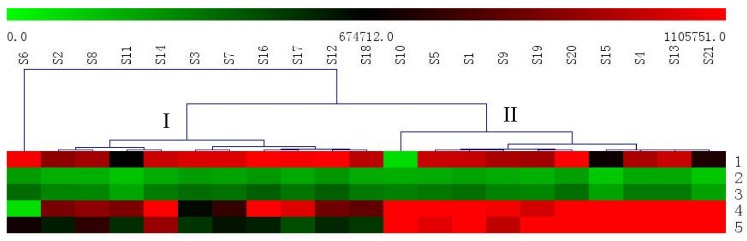
Heat map of the relative concentrations of the five major analytes in all the assessed samples. The S1–21 in the figure represents the samples of different regions of Henan Province. The numbers of 1–5 in the figure represents the five major analytes, including *β*-ecdysterone (1); 25-S inokosterone (2); 25-R inokosterone (3); ginsenoside R_0_ (4); and chikusetsusaponin IVa (5).

Hierarchical clustering analysis (HCA) is a statistical method for finding relatively homogeneous sample groups based on selected characteristics. The approach has been widely used for species authentication, origin discrimination, and quality evaluation of traditional Chinese medicines [49,50]. To analyze the samples from Henan, the data from 21 batches of raw RAB were imported into the MultiExperiment Viewer (MeV) software (Dana-Farber Cancer Institute, Boston, MA, USA). Trends with regards to the relative concentrations of the target analytes in the samples were visualized using a heat map. As shown in Figure 4, the analytes content of the samples exhibited a red color on the heat map, indicating that these samples contained different levels of the five analytes. From green to red, the heat map colors represent the relative content of the five analytes in all the assessed samples. The red represents the relative highest content. With the green color deepening, the relative content decreased. First, S6 are distinctly different in Heshan, which is the reason for the maximum content of *β*-ecdysterone and reduced content of triterpenoid saponins. Secondly, it was evident that the other RAB samples were clearly clustered into two groups: I (Zhengzhou, Luoyang, Kaifeng, Nanyang, Zhoukou, Jinshui, Longting, Gulou, Lankao, and Xiangfu) and II (Qinyang, Wushe, Wenxian, Xiayi, Mengxian, Boai, Macun, Jiefang, and Shanyang Hongqi). This means that the five major analytes were significantly different in different RABs. As shown in Figure 4, the total content of the phytoecdysones and triterpenoid saponins in Group II had a red color on the heat map, indicating that II group samples contained higher levels of the analytes and were of better quality medicinal materials compared with the samples from other regions. These differences are probably due to some factors, such as intrinsic factors, including plant origin and genetic variation, and extrinsic factors, including the season (different climatic), geography (soil and minerals conditions), harvest time, storage conditions, and environmental pollution. Finally, the information from Table 4 shows that there is different content in different batches of Chinese medicine from the Henan Province. These all suggested that each collection procedure should be standardized in the future to ensure the quality of RAB. 

#### 2.5.2. Principal Component Analysis

**Figure 5:**
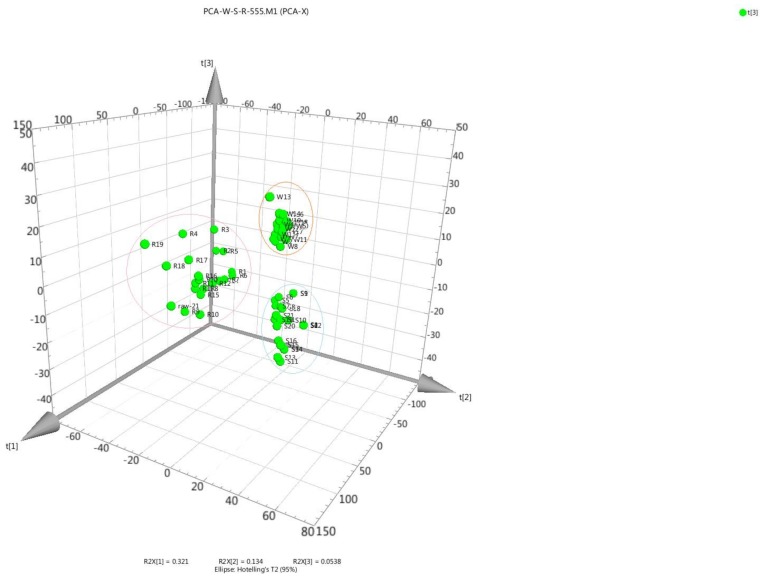
Three-dimensional principal component analysis (PCA) plots in 21 batches of raw RAB (RRAB), wine RAB (WRAB), and salt RAB (SRAB). Letters on the figure denote the processed type investigated: (R) RRAB, (W) WRAB, and (S) SRAB.

Principal component analysis (PCA) is a widely used method that provides an interpretable overview of the main information in numerical datasets in a multivariate space [51]. To analyze the differences between the raw, wine-processed, and salt-processed groups, the data from the three groups were imported into the SIMCA-P 13 software package. The concentrations of the RRAB, WRAB, and SRAB were calculated in the three-dimensional space. According to the three dimensional (3D) score plots (Figure 5), RRAB, WRAB, and SRAB were reasonably well separated on the 3D coordinates for the three different processed methods with dramatic differences between them, indicating that variations in the chemical content of the three different processed methods were remarkable. The samples from the different processed methods were classified into three sub-clusters by PCA. These results suggested that RRAB, WRAB, and SRAB may possess different qualities, efficacies and indications. The analysis of five major analytes was suitable for the quality control of RAB. 

In fact, the importance of the chemometric method has been highlighted based on their wide use in the quality control of herbal medicines [52,53,54]. In this study, PCA coupled with boxplots indicated that the processing methods result in significant differences in quality and illustrated that the salt and wine-processed RAB significantly alters chemical components compared to the raw RAB. These two methods play an important role and combined with UHPLC-MS/MS, the PCA analytical approaches can be used to discriminate different plant origin, productive processes, cultivation pattern, and other factors that may affect the quality of RAB. This method can enhance the quality evaluation of RAB and ensure efficacious and safe use, which can provide a completely new way for quick and accurate analyses of herbal medicines.

## 3. Materials and Methods 

### 3.1. Chemicals and Reagents 

HPLC grade acetonitrile was obtained from Thermo Fisher Scientific, America. HPLC grade acetic acid was obtained from Dimka Pure (Richmond Hill, NY, USA). Other reagent solutions and chemicals were of analytical grade. Chemical standards of *β*-ecdysterone, ginsenoside R_0_, chikusetsusaponin IVa and glycyrrhizin (IS) were obtained from Chengdu Must Bio-Technology Co., Ltd., Chengdu, China. For this study, 25-R inokosterone and 25-S inokosterone were isolated and identified from the roots of RAB by our laboratory. The purity of each compound was more than 98%, which was determined by HPLC analysis. 

Twenty-one batches of RAB were collected from different regions of Henan Province. The wine-processing procedure and salt-processing procedure were performed according to the 0213 general rules of processing in Chinese pharmacopeia 2015 edition. The botanical origins of the plants were identified as RAB by Prof. Lianjie Su and the voucher specimens (accession number, PC2012216019) were deposited at Heilongjiang University of Chinese Medicine, Harbin, China.

### 3.2. Preparation of Sample Solutions

All the RAB samples were pulverized and sieved (60 mesh). A total of 0.5 g of the powdered sample was immersed into a methanol/water solvent in a flask, before being ultrasonicated in a water bath. Three parameters were investigated to optimize the extraction conditions. Different solvent volumes (25, 50, and 75 mL), (30, 40, 60, and 80 min) and extraction solvent (25, 50, 75, and 100% methanol/water) were employed for optimization of the extraction rates. An aliquot (5 μL) of the supernatant solution was injected into UHPLC–MS/MS for analysis.

### 3.3. UHPLC–MS/MS Analysis Conditions

Chromatographic analysis was performed in an ultra-high performance liquid chromatography (Thermo Scientific^TM^, Vanquish^TM^, (Waltham, MA, USA), using a Thermo Hypersil GOLD (Waltham, MA, USA) C_18_ column (100 mm × 2.1 mm, 1.9 μm). The column temperature was maintained at 35 °C. The mobile phase was composed of acetonitrile and water (both containing 0.3% acetic acid), with a gradient elution: 0–6 min and 83% water; 6–8 min and 5–84% water; and 8–10 min and 5% water. The flow rate of the mobile phase was 0.3 mL/min, while the injection volume was 5 μL.

Mass spectrometric detection was carried out using a Thermo TSQ QUANTIS triple quadrupole mass spectrometer using electron spray ionization (ESI) source operated in negative ion mode. The parameters in the source were set as follows: sheath gas of 40 Arb; aux gas of 10 Arb; ion transfer tube temp of 325 °C; and vaporizer temperature of 350 °C. 

### 3.4. Validation of the Method 

For calibration, the linearity was used to describe the relationship of the analyte concentrations and detector response based on peak area ratio of analytes to IS. The lowest concentration of the working solution for calibration use was diluted with methanol/water to a series of appropriate concentrations. Fresh calibration standards were prepared for each day of analysis during the validation. LOD and LOQ were determined at a signal-to-noise ratio (S/N) of about 3 and 10, respectively. The precision was evaluated by analyzing the standard solutions containing the five standard analytes six times. For intra-day variability test, the individual sample solution was analyzed six times within one day (*n* = 6), while the inter-day reproducibility was determined with six individual sample solutions for three consecutive days (*n* = 6). To confirm the repeatability, six different sample solutions prepared from the same sample (sample 5) were analyzed and variations were expressed by RSD. For stability investigation, one of the sample solutions mentioned above was stored at 25 °C and analyzed at 0, 4, 8, 16, 32, and 48 h. A recovery test was used to evaluate the accuracy of this method. The spiked samples were subsequently extracted, processed and quantified in accordance with the methods mentioned above. The extraction recoveries of the analytes were investigated at three QC levels. Briefly, the sample was spiked with known amounts of the standard analytes at low, medium and high concentrations for the five analytes. These were thoroughly mixed, before being extracted and analyzed under optimized conditions. The average recoveries were calculated by the formulae: recovery (%) = (amount found−original amount)/amount spiked × 100% and RSD (%) = (SD/mean) × 100%.

### 3.5. Qualitative and Quantitative Analysis

The identification of five major analytes (*β*-ecdysterone, 25-R inokosterone, 25-S inokosterone, ginsenoside R_0_ and chikusetsusaponin IVa) was carried out by comparing the UHPLC retention time of target peaks and the characteristics of their protonated ions with those of the standards by UHPLC–MS/MS in negative ion mode. Quantification was performed by plotting the peak-area ratio of the five analytes to IS against their concentrations.

### 3.6. Applications for Different Batches of Raw, Wine-Processed, and Salt-Processed Products

The established UHPLC–MS/MS approach was applied for the simultaneous quantification of *β*-ecdysterone, 25-R inokosterone, 25-S inokosterone, ginsenoside R_0_ and chikusetsusaponin IVa in 21 batches of raw, wine-processed, and salt-processed products. The contents of the five analytes in 21 different batches of raw, wine-processed, and salt-processed RAB were determined.

### 3.7. Data Processing

All the experiments were performed at least in triplicate with constant results. Differences among groups were considered significant at *p* < 0.05. The heat map and HCA were made using MultiExperiment Viewer (MeV) software (Dana-Farber Cancer Institute, Boston, MA, USA) with z-score normalization. Principal component analysis (PCA) was performed by SIMCA 13.0 software (Umetrics, Umeå, Sweden). The boxplot were charted by Origin 8 (OriginLab, Northampton, MA, USA).

## 4. Conclusions

In this work, a simple, sensitive and reliable UHPLC-MS/MS method for simultaneous quantification of five analytes in 21 batches of raw, wine-processed, and salt-processed RAB was developed and validated. The study suggested that a combination of UHPLC-MS/MS and chemometric methods could discriminate raw, wine-processed, and salt-processed samples under different processing conditions. The results of this study proved that the content of salt-processed RAB is better than that of raw products and wine-processed products. Notably, the bioactive analytes after salt-processing will contribute to the reinforcement of liver and kidney nourishment. The results show that the study provides a theoretical basis for the salt-processed RAB nourishing liver and kidney and strengthening bones and tendons. In summary, this study has established an efficient analytical method for the quality control of RAB and has also provided a valuable reference for the quality assessment of other herbal medicines. 

## Figures and Tables

**Table 1:** Precursor/product ion pairs and parameters for selected reaction monitoring (SRM) of the five analytes and internal standard.

Analytes	t_R_ (min)	[M − H]^−^	SRM Transitions (Precursor→Product)	Collision Energy (eV)
*β-*ecdysterone	2.85	479.519	479.519→319.0	28.54
25-R inokosterone	3.24	479.519	479.519→319.0	28.54
25-S inokosterone	3.52	479.519	479.519→319.0	28.54
ginsenoside R_0_	8.65	955.745	955.745→793.5	55.00
chikusetsusaponin IVa	8.75	793.678	793.678→631.4	47.04
glycyrrhizin (I.S.)	8.52	255.207	255.207→119.0	25.43

**Table 2:** Calibration curves, correlation coefficients, linearity ranges, limit of detection (LOD) and limit of quantity (LOQ) data of the five investigated analytes.

Analytes	Calibration Curves	r^2^	Linearity Ranges (mg/mL)	LOD (mg/mL)	LOQ (mg/mL)
*β-*ecdysterone	y = 0.0545x − 0.0558	0.9993	1.90–494.0	0.365	1.085
25-S inokosterone	y = 0.3174x − 0.3118	0.9995	1.35–270.0	0.332	0.995
25-R inokosterone	y = 0.3814x − 0.5633	0.9993	1.75–315.0	0.499	1.485
ginsenoside R_0_	y = 0.4588x − 0.4343	0.9994	2.10–210.0	0.325	0.955
Chikusetsusaponin IVa	y = 0.6858x − 0.7556	0.9994	1.85–185.0	0.369	1.105

**Table 3:** Precision, repeatability, stability, and recovery of the five investigated analytes (overall intra-day and inter-day variations (RSD), %, *n* = 6).

Analytes	Precision		Repeatability	Stability	Recovery (%, mean/RSD, *n* = 3)		
	Intra-Day	Inter-Day			Low	Medium	High
*β-*ecdysterone	1.57	2.25	2.95	2.68	98.91 (0.01)	99.31 (0.07)	99.29 (0.07)
25-S inokosterone	2.46	1.51	2.25	2.54	98.98 (0.47)	101.17 (0.85)	98.58 (1.86)
25-R inokosterone	1.83	1.72	2.58	2.97	101.48 (0.14)	98.68 (0.42)	99.81 (0.90)
ginsenoside R_0_	2.34	1.68	2.16	2.58	101.09 (0.28)	100.57 (0.72)	100.89 (0.15)
Chikusetsusaponin IVa	1.78	3.00	3.00	2.63	101.41 (0.23)	99.75 (0.84)	99.97 (0.78)

**Table 4:** Contents of five analytes in 21 batches of raw, wine-processed, and salt-processed samples.

No.	Region or Pharmacy (Specimen No.)	Content of Investigated Components (*n* = 3, mg/g ± SD)														
		*β-*Ecdysterone (1)			25-R Inokosterone (2)			25-S Inokosterone (3)			Ginsenoside R_0_ (4)			Chikusetsusaponin IVa (5)		
		RRAB	WRAB	SRAB	RRAB	WRAB	SRAB	RRAB	WRAB	SRAB	RRAB	WRAB	SRAB	RRAB	WRAB	SRAB
S1	Wenxian, Henan (NWH2016-01)	12.778 ± 0.318	31.214 ± 0.198	88.051 ± 0.410	0.636 ± 0.006	1.453 ± 2.545	4.667 ± 0.791	0.880 ± 0.009	1.935 ± 0.025	6.153 ± 0.084	0.740 ± 0.024	4.396 ± 0.020	11.221 ± 0.009	0.956 ± 0.010	2.943 ± 0.001	13.864 ± 0.226
S2	Zhengzhou, Henan (NZH2016-02)	26.493 ± 0.167	32.577 ± 0.002	81.066 ± 0.387	1.153 ± 0.034	1.477 ± 0.001	4.117 ± 0.097	1.458 ± 0.019	1.897 ± 0.029	41.570 ± 0.716	0.400 ± 0.002	3.880 ± 0.024	12.268 ± 0.068	0.444 ± 0.002	1.905 ± 0.007	13.608 ± 0.448
S3	Zhoukou, Henan (NZH2016-03)	38.670 ± 0.194	38.554 ± 0.002	81.746 ± 0.335	1.680 ± 0.002	1.798 ± 0.073	4.417 ± 0.030	2.216 ± 0.016	2.308 ± 0.003	5.852 ± 0.011	2.232 ± 0.002	3.033 ± 0.076	9.5488 ± 0.006	1.319 ± 0.002	1.784 ± 0.003	12.519 ± 0.019
S4	Jiefang, Henan (NJH2016-04)	23.406 ± 0.123	30.819 ± 0.017	50.605 ± 0.404	1.083 ± 0.002	1.423 ± 0.003	2.982 ± 0.560	1.437 ± 0.036	1.844 ± 0.003	3.979 ± 0.096	7.520 ± 0.005	5.866 ± 0.001	12.925 ± 0.108	3.250 ± 0.197	4.525 ± 0.014	16.179 ± 0.006
S5	Wushe, Henan (NWH2016-05)	26.280 ± 0.262	35.231 ± 0.021	92.555 ± 0.613	1.141 ± 0.067	1.516 ± 0.003	4.677 ± 0.061	1.554 ± 0.002	2.045 ± 0.011	6.177 ± 0.085	6.108 ± 0.030	5.323 ± 0.023	11.986 ± 0.049	1.715 ± 0.001	3.131 ± 0.004	13.634 ± 0.017
S6	Heshan, Henan (NHH2016-06)	14.076 ± 0.229	43.287 ± 0.025	77.559 ± 0.796	0.727 ± 0.004	1.948 ± 0.001	4.149 ± 0.014	0.986 ± 0.010	2.495 ± 0.001	5.545 ± 0/243	0.406 ± 0.002	0.580 ± 0.001	12.358 ± 0.383	0.483 ± 0.01	2.438 ± 0.002	13.716 ± 0.09
S7	Jinshui, Henan (NJH2016-07)	11.332 ± 0.015	39.833 ± 0.020	70.805 ± 0.042	0.599 ± 0.013	1.754 ± 0.001	3.794 ± 0.018	0.812 ± 0.004	2.271 ± 0.003	4.892 ± 0.196	0.385 ± 0.005	3.604 ± 0.008	6.401 ± 0.054	0.504 ± 0.006	2.078 ± 0.017	6.0731 ± 0.022
S8	Luoyang, Henan (NLH2016-08)	24.191 ± 0.036	36.948 ± 0.003	68.983 ± 0.428	1.115 ± 0.002	1.578 ± 0.004	3.789 ± 1.459	1.472 ± 0.003	2.035 ± 0.003	4.952 ± 0.144	6.654 ± 0.014	4.394 ± 0.001	13.239 ± 3.978	1.322 ± 0.008	2.544 ± 0.002	13.989 ± 0.004
S9	Xiayi, Henan (NXH2016-09)	25.973 ± 0.215	35.182 ± 0.055	67.382 ± 0.603	1.230 ± 0.067	1.594 ± 0.002	29.085 ± 0.013	1.601 ± 0.006	1.984 ± 0.006	4.955 ± 0.049	12.930 ± 0.011	4.867 ± 0.023	22.730 ± 0.091	1.611 ± 0.007	3.099 ± 0.034	24.632 ± 0.006
S10	Qinyang, Henan (NQH2016-10)	16.420 ± 0.106	4.202 ± 0.024	79.573 ± 0.177	0.806 ± 0.034	1.779 ± 0.001	4.242 ± 0.472	1.080 ± 0.002	2.354 ± 0.005	5.639 ± 0,.181	6.467 ± 0.108	9.142 ± 0.007	28.838 ± 0.164	2.272 ± 0.008	6.523 ± 0.034	30.468 ± 0.503
S11	Kaifeng, Henan (NKH2016-11)	30.524 ± 0.013	21.687 ± 0.204	92.820 ± 0.568	1.397 ± 0.004	1.051 ± 0.001	4.966 ± 0.033	1.829 ± 0.004	1.359 ± 0.079	6.458 ± 0.035	4.089 ± 0.030	3.540 ± 0.042	4.935 ± 0.004	1.142 ± 0.002	1.616 ± 0.001	7.0181 ± 0.007
S12	Lankao, Henan (NLH2016-12)	12.690 ± 0.101	37.268 ± 0.031	56.059 ± 0.068	0.646 ± 0.002	1.712 ± 0.057	3.267 ± 0.004	0.882 ± 0.001	2.208 ± 0.025	4.335 ± 0.019	1.310 ± 0.001	3.470 ± 0.004	16.011 ± 0.037	0.728 ± 0.001	1.656 ± 0.021	10.215 ± 0.016
S13	Shanyang, Henan (NSH2016-13)	28.123 ± 0.053	29.411 ± 0.175	44.903 ± 0.086	1.255 ± 0.043	1.315 ± 0.001	2.648 ± 0.047	1.635 ± 0.001	1.715 ± 0.005	3.567 ± 0.109	10.360 ± 0.001	6.113 ± 0.001	6.812 ± 0.153	2.427 ± 0.010	4.202 ± 0.147	4.517 ± 0.07
S14	Nanyang, Henan (NNH2016-14)	7.6519 ± 0.064	26.536 ± 0.157	68.005 ± 0.099	0.446 ± 0.006	1.173 ± 0.001	3.612 ± 0.581	0.621 ± 0.002	1.562 ± 0.008	4.790 ± 0.014	1.100 ± 0.001	4.006 ± 0.003	17.141 ± 0.070	0.700 ± 0.001	2.133 ± 0.024	8.525 ± 0.002
S15	Macun, Henan (NMH2016-15)	16.737 ± 0.003	24.189 ± 0.034	78.139 ± 0.497	0.768 ± 0.014	1.057 ± 0.057	3.823 ± 0.289	1.078 ± 0.001	1.468 ± 0.010	5.214 ± 0.015	10.151 ± 0.08	7.249 ± 0.004	17.032 ± 0.052	2.862 ± 0.008	4.830 ± 0.007	16.423 ± 0.008
S16	Longting, Henan (NLH2016-16)	23.450 ± 0.014	39.351 ± 0.050	59.081 ± 0.377	1.051 ± 0.015	1.726 ± 0.001	3.246 ± 0.003	1.414 ± 0.001	2.272 ± 0.014	4.353 ± 0.109	2.905 ± 0.135	4.513 ± 0.003	9.875 ± 0.227	1.212 ± 0.010	1.750 ± 0.006	3.0279 ± 0.001
S17	Gulou, Henan (NGH2016-17)	15.539 ± 0.174	30.572 ± 0.003	67.816 ± 0.331	0.716 ± 0.003	1.279 ± 0.003	3.343 ± 0.001	0.995 ± 0.004	1.722 ± 0.09	4.628 ± 0.0119	2.034 ± 0.206	3.646 ± 0.006	5.127 ± 0.042	1.005 ± 0.001	1.253 ± 0.001	6.234 ± 0.029
S18	Xiangfu, Henan (NXH2016-18)	30.854 ± 1.209	33.943 ± 0.003	96.379 ± 0.332	1.304 ± 0.003	1.513 ± 0.046	4.800 ± 0.020	1.768 ± 0.001	1.991 ± 0.006	6.297 ± 0.198	5.200 ± 0.147	3.600 ± 00.004	11.494 ± 0.029	1.797 ± 0.001	1.635 ± 0.004	5.432 ± 0.001
S19	Mengxian, Henan (NMH2016-19)	22.038 ± 0.009	26.264 ± 0.013	83.783 ± 0.210	1.022 ± 0.015	1.159 ± 0.001	4.127 ± 0.272	1.343 ± 0.005	1.536 ± 0.004	5.558 ± 0.073	5.119 ± 0.040	3.545 ± 1.156	13.331 ± 0.006	2.164 ± 0.001	2.846 ± 0.005	10.735 ± 0.001
S20	Boai, Henan (NBH2016-20)	10.088 ± 0.009	38.828 ± 0.003	86.465 ± 1.138	0.536 ± 0.002	1.695 ± 0.001	4.360 ± 0.023	0.743 ± 0.001	2.202 ± 0.086	5.632 ± 0.074	2.009 ± 0.011	5.517 ± 0.023	21.533 ± 0.012	1.157 ± 0.001	4.126 ± 0.015	15.114 ± 0.001
S21	Hongqi, Henan (NHH2016-21)	18.976 ± 1.070	27.053 ± 0.020	35.614 ± 2.982	0.833 ± 0.020	1.141 ± 0.001	2.210 ± 0.083	1.152 ± 0.02	1.569 ± 0.012	3.121 ± 0.068	11.546 ± 0.037	6.552 ± 0.016	13.514 ± 0.001	3.277 ± 0.002	4.511 ± 0.179	8.721 ± 0.017

## Supplementary Materials

The Supplementary Materials are available online.
