# Supplementary material for: UHPLC-MS/MS Quantification Combined with Chemometrics for Comparative Analysis of Different Batches of Raw, Wine-Processed, and Salt-Processed Radix Achyranthis Bidentatae
Source: Molecules. 2018 Mar 26;23(4):758. doi: 10.3390/molecules23040758 (PMC6017346; doi:10.3390/molecules23040758)
Supplement: Supplementary file 1 [file molecules-23-00758-s001.pdf]

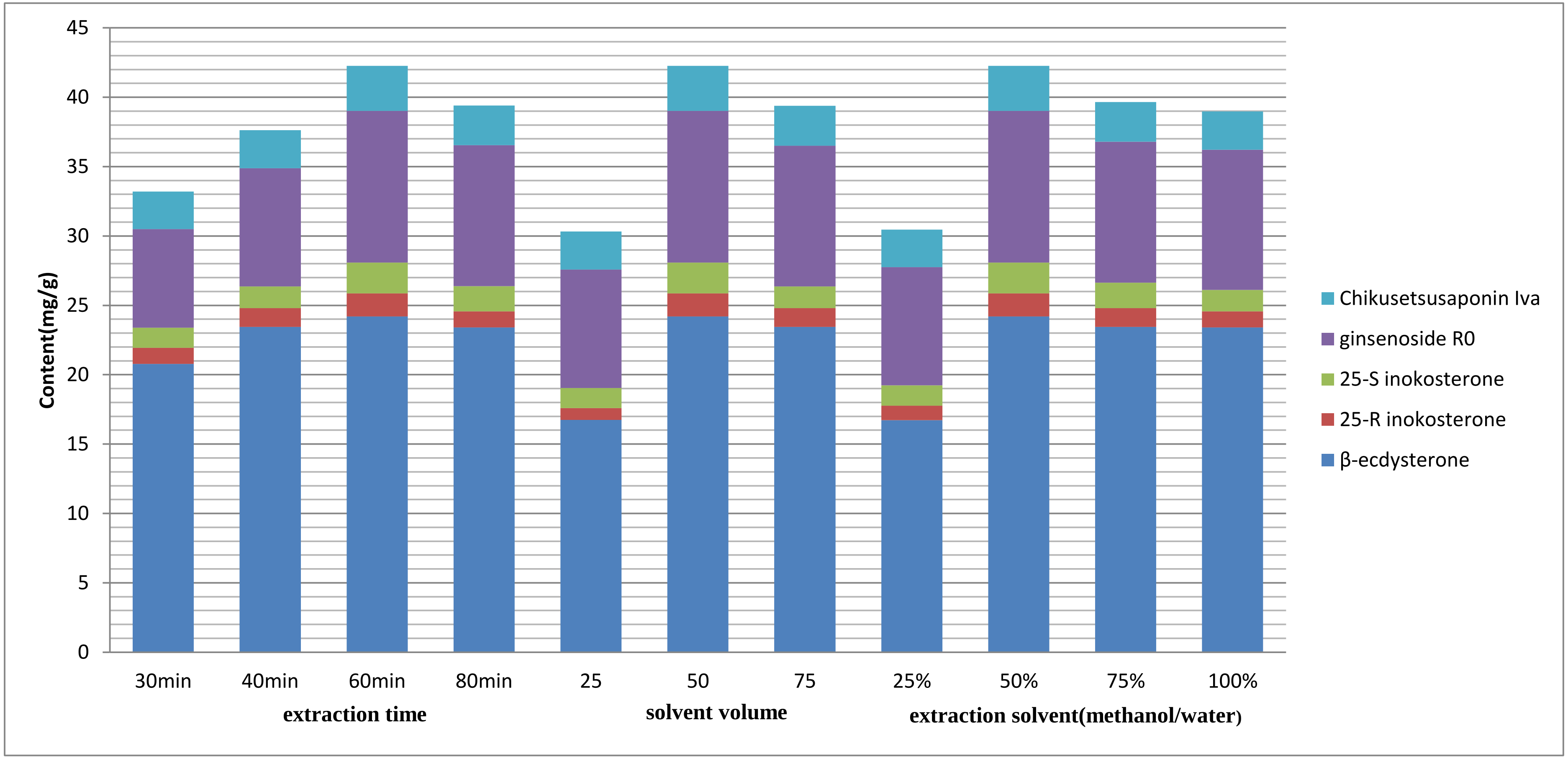

Figure1S，Effects of extraction solvent volume, time, repetition on the extraction efficiency of the target analytes in RAB collected from Henan (S20), China. 1
